# Improved characterization of sub-centimeter enhancing breast masses on MRI with radiomics and machine learning in BRCA mutation carriers

**DOI:** 10.1007/s00330-020-06991-7

**Published:** 2020-06-27

**Authors:** Roberto Lo Gullo, Isaac Daimiel, Carolina Rossi Saccarelli, Almir Bitencourt, Peter Gibbs, Michael J. Fox, Sunitha B. Thakur, Danny F. Martinez, Maxine S. Jochelson, Elizabeth A. Morris, Katja Pinker

**Affiliations:** 1 Department of Radiology, Breast Imaging Service, Memorial Sloan Kettering Cancer Center, 300 E 66th Street, New York, NY 10065 USA; 2 Department of Radiology, Memorial Sloan Kettering Cancer Center, 300 E 66th Street, New York, NY 10065 USA; 3 Sloan Kettering Institute, Memorial Sloan Kettering Cancer Center, Mortimer B. Zuckerman Research Center, 417 E 68th Street, New York, NY 10065 USA; 4 Department of Medical Physics, Memorial Sloan Kettering Cancer Center, 1275 York Ave, New York, NY 10065 USA; 5 Department of Biomedical Imaging and Image-Guided Therapy, Molecular and Gender Imaging Service, Medical University of Vienna, Waehringer Guertel 18-20, 1090 Wien, Austria

**Keywords:** Machine learning, Breast neoplasms, Artificial intelligence, Magnetic resonance imaging

## Abstract

**Objectives:**

To investigate whether radiomics features extracted from MRI of BRCA-positive patients with sub-centimeter breast masses can be coupled with machine learning to differentiate benign from malignant lesions using model-free parameter maps.

**Methods:**

In this retrospective study, BRCA-positive patients who had an MRI from November 2013 to February 2019 that led to a biopsy (BI-RADS 4) or imaging follow-up (BI-RADS 3) for sub-centimeter lesions were included. Two radiologists assessed all lesions independently and in consensus according to BI-RADS. Radiomics features were calculated using open-source CERR software. Univariate analysis and multivariate modeling were performed to identify significant radiomics features and clinical factors to be included in a machine learning model to differentiate malignant from benign lesions.

**Results:**

Ninety-six BRCA mutation carriers (mean age at biopsy = 45.5 ± 13.5 years) were included. Consensus BI-RADS classification assessment achieved a diagnostic accuracy of 53.4%, sensitivity of 75% (30/40), specificity of 42.1% (32/76), PPV of 40.5% (30/74), and NPV of 76.2% (32/42). The machine learning model combining five parameters (age, lesion location, GLCM-based correlation from the pre-contrast phase, first-order coefficient of variation from the 1st post-contrast phase, and SZM-based gray level variance from the 1st post-contrast phase) achieved a diagnostic accuracy of 81.5%, sensitivity of 63.2% (24/38), specificity of 91.4% (64/70), PPV of 80.0% (24/30), and NPV of 82.1% (64/78).

**Conclusions:**

Radiomics analysis coupled with machine learning improves the diagnostic accuracy of MRI in characterizing sub-centimeter breast masses as benign or malignant compared with qualitative morphological assessment with BI-RADS classification alone in BRCA mutation carriers.

**Key Points:**

• *Radiomics and machine learning can help differentiate benign from malignant breast masses even if the masses are small and morphological features are benign.*

*• Radiomics and machine learning analysis showed improved diagnostic accuracy, specificity, PPV, and NPV compared with qualitative morphological assessment alone.*

**Electronic supplementary material:**

The online version of this article (10.1007/s00330-020-06991-7) contains supplementary material, which is available to authorized users.

## Introduction

Women who inherit BRCA1 and BRCA2 mutations lack tumor suppressor proteins that repair damaged DNA [1]. These women have an increased risk of developing breast cancer at a younger age compared with women who do not have these mutations. MRI is the most sensitive imaging modality for breast cancer detection and therefore, the American Cancer Society and the American College of Radiology recommend yearly mammography in BRCA mutation carriers starting at age 30 years and yearly MRI beginning at age 25 [2–7].

A significant proportion (45%) of BRCA1-related cancers are seen only on MRI [8] where they tend to be cellular with round pushing margins rather than scirrhous with irregular infiltrating margins as seen in other breast cancers. Therefore, early/small tumors may not exhibit classic malignant features but rather may exhibit a benign imaging appearance [9]. As these cancers are also more likely to be high grade and frequently triple negative (hormone receptor and HER-2 negative), the threshold for the recommendation of a biopsy should be low [10, 11]. Prior studies [12, 13] showed how benign morphology is common in invasive cancers of less than 5 mm in diameter regardless of BRCA mutation status and suggested that all masses representing an interval change as well as lesions increasing in size should lead to a biopsy. Unfortunately, BRCA carriers are also more prone to developing benign tumors of the breast [14, 15], resulting in numerous benign biopsies during their life unless prophylactic mastectomy is performed.

To avoid missing significant cancers as well as exposing women to unnecessary biopsies, additional tools to help discriminate benign from malignant lesions should be used to predict the likelihood of malignancy. Radiomics analysis involves the quantitative assessment of the pixel intensity arrangement within specific regions of interest (ROIs) and extracts quantitative features that can be used for further disease characterization. Initial results in women at average risk of breast cancer indicate that radiomics analysis and machine learning (ML) are of value in distinguishing benign and malignant small breast masses [16].

The purpose of our study was to investigate whether radiomics features extracted from MRI of BRCA-positive patients with sub-centimeter breast masses can be coupled with machine learning to differentiate benign from malignant lesions using model-free parameter maps.

## Materials and methods

### Study population

This was a retrospective Health Insurance Portability and Accountability Act–compliant study conducted at Memorial Sloan Kettering Cancer Center. The study was approved by the Institutional Review Board (protocol number 19-119) and the need for written informed consent was waived.

A review of the Department of Radiology database was performed to identify consecutive patients with genetic testing results available and who had an MRI from November 2013 to February 2019 that led to a biopsy or a short-term follow-up. We identified 430 patients. Our inclusion criteria were as follows: BRCA 1– or BRCA 2–positive patients; breast masses with the longest diameter ≤ 10 mm; and BI-RADS 3, 4, or 5 on MRI further assessed with follow-up or vacuum-assisted breast biopsy (MRI or ultrasound-guided) yielding benign or malignant histology. Findings described as non-mass enhancements on MRI were not included. We excluded patients with mutations other than BRCA 1 and 2 and those with a follow-up of less than 2 years when biopsy was not performed (BI-RADS 3 and BI-RADS 4 when target was not visualized at the time of biopsy).

### Breast MRI technique

Breast MRI was performed on either a 1.5-T or a 3-T magnet (Sigma; GE) using an 8-channel or 16-channel dedicated surface breast coil. The imaging sequences are included in Table 1.

**Table 1:** Summary of imaging sequences and acquisition parameters used for the study

MR sequences	Acquisition parameters
Axial fat-suppressed 2D T2-weighted imaging	TR, 5000–6000 ms; TE, 90–110 ms; refocusing flip angle, “auto”; slice thickness, 3 mm; gap, 0 mm; field of view, 34–38 cm; matrix size, 320 × 320; bandwidth, 125 kHz for 1.5 T and 83 kHz for 3.0 T; parallel imaging, “ASSET”
Axial non-fat-suppressed 3D T1-weighted imaging	
Axial fat-suppressed 3D T1-weighted imaging using a Volume Image Breast Assessment (VIBRANT) gradient echo. One sequence before and 3 sequences after intravenous administration of a gadolinium-based contrast agent	TR, 4–4.5 ms; TE, 2.1 ms; flip angle, 10°; bandwidth, 62 kHz; field of view, 34–38 cm; matrix size, 320 × 192 (for 1.5 T) and 300 × 300 (for 3.0 T); slice thickness, 1.1 mm; gap, 0 mm; parallel imaging, “ASSET”
Axial DWI using single-shot with echo-planar imaging (EPI)	2 b-values (b = 0, 800); TR, 6000 ms; TE, “minimum”; flip angle, 90°; field of view, 34–38 cm, matrix size, 128 × 128 (for 1.5 T), 256 × 256 (for 3 T); fat suppression, “special”; dual shims, “on”; slice thickness, 4–5 mm; parallel imaging, “ASSET”
ADC mapping available in 65 lesions	

*ASSET*, array spatial sensitivity encoding technique; *TR*, repetition time; *TE*, echo time

### Imaging assessment by radiologists

All images were independently assessed by two dedicated fellowship-trained breast radiologists in one session (R1: R.L., and R2: I.D., both with 4 years of experience in breast imaging and interpreting breast MRI) blinded to the final histopathological diagnoses and prior or subsequent conventional and MRI imaging. For each lesion, the following morphological features were assessed according to the BI-RADS lexicon on post-contrast-enhanced T1-weighted images: lesion shape, margin, and internal enhancement characteristics. Readers also assigned a BI-RADS classification. Lesion size was measured as the single largest diameter. On T2-weighted and DW images, signal intensity, morphology, background parenchymal enhancement (BPE), and fibroglandular tissue (FGT) for each breast were also assessed. Time–intensity kinetic curve analysis (signal enhancement in relation to time after contrast injection) was performed on a dedicated workstation with a commercially available computer-aided diagnosis system (OsiriX, OsiriX Foundation) by R1. The reader qualitatively measured the kinetic curve pattern described as washout, plateau, or persistent, according to the BI-RADS lexicon. The location of lesions within the breast (anterior, middle, or posterior depth) was also assessed by R1.

After independent review was conducted, the cases in which there was disagreement between the two readers were re-reviewed in consensus to generate an overall consensus assessment.

### Reference standard

Preferentially, histopathology was used as the reference standard established by either image-guided needle biopsy or surgery. In two patients who had benign high-risk lesions on biopsy, the histological report from the surgical biopsy was recorded to confirm the benign nature of the lesion. When biopsy was not performed, stability of more than 2 years on follow-up MRI was considered benign.

### Radiomics analysis

Digital Imaging and Communications in Medicine (DICOM) images from the DCE-MRI and non-contrast-enhanced T1-weighted MRI were loaded into the open-source image processing tool OsiriX. Both radiologists reviewed the images in consensus before delineating the ROIs and R1manually delineated the ROIs, tracing the borders of each lesion to include the entire enhancing lesion.

Given the small size of the lesions sampled yielding a small number of pixels per slice, an in-house code written in MATLAB (The MathWorks, Inc.) was used to input the ROIs into the open-source CERR software environment (freely available through GitHub) which calculated the radiomics features [17]. Data was reduced to 16 gray levels and only an interpixel distance of one was considered (for small lesions, higher interpixel distances are not appropriate and would reduce counting statistics drastically). CERR analysis resulted in 102 radiomics features sub-divided into six categories: 22 first-order features, 26 features based on the gray level co-occurrence matrix (GLCM), 16 features based on the run length matrix (RLM), 16 features based on the size zone matrix (SZM), 17 features based on the neighborhood gray level dependence matrix, and 5 features based on the neighborhood gray tone difference matrix. Since patients were scanned at either 1.5 T (27 benign cases and 17 malignant cases) or 3 T (49 benign cases and 23 malignant cases), ComBat harmonization (Supplemental Info A1) was employed prior to statistical analysis to remove center effects [18].

Univariate analysis was initially performed to select significant radiomics features able to differentiate between benign and malignant lesions. An AUC cutoff of ≥ 0.65 was used to reduce the number of features of interest. Correlation analysis was then employed to further remove redundant features. For any significant correlations in which the Spearman rank correlation coefficient > 0.9, the feature with the lowest AUC was removed from consideration. This resulted in a more manageable number of features for subsequent multivariate modeling. Using a fine Gaussian support vector machine, perfect separation of benign and malignant cases was obtained. To limit data overfitting, a fivefold cross-validation was employed to develop a robust ML model which should produce similar results for new data.

### Statistical analysis

Statistical analysis was conducted using SAS (version 9.4, SAS Institute). Continuous variables were summarized using means (± standard deviation) and medians (range); categorical variables were summarized using proportions. Univariate analysis using the chi-square test or Fisher’s exact test was performed to assess associations between the imaging parameters (from independent and consensus assessment) with disease status (malignant vs. benign). *p* values < 0.05 were considered significant. To determine inter-observer agreement, weighted Cohen’s *κ* was used to assess ordinal parameters, while simple Cohen’s *κ* was used to assess the inter-reader agreement for nominal parameters.

For radiomics data, statistical analysis was performed using SPSS (version 25, IBM Corp.) and MATLAB (R2017b, The MathWorks, Inc.). Univariate analysis was performed to identify radiomics features that were significantly different between malignant and benign lesions. Since the number of patients was not large (especially in the malignant cohort), normality in the malignant and benign cohort distributions was tested using the Shapiro–Wilk test and Q-Q plots. For a minority (21/102) of normally distributed features, a two-tailed independent *t* test was used to determine the significant features. For the majority of non-normally distributed features (81/102), the Mann–Whitney *U* test for two independent samples was used to determine the significant features.

Clinical factors considered as potential predictors of malignancy (age, BRCA status, menopausal status, and lesion location) were assessed for statistically significant associations with disease status using the Mann–Whitney *U* test (for age) and the Pearson chi-square test (for all other clinical factors). Significant clinical factors were incorporated into multivariate modeling along with significant radiomics features to produce a robust ML model for discriminating between benign and malignant lesions. All ML modelling was performed using a predefined Gaussian support vector machine.

## Results

### Patient population and breast lesion characteristics

**Fig. 1:**
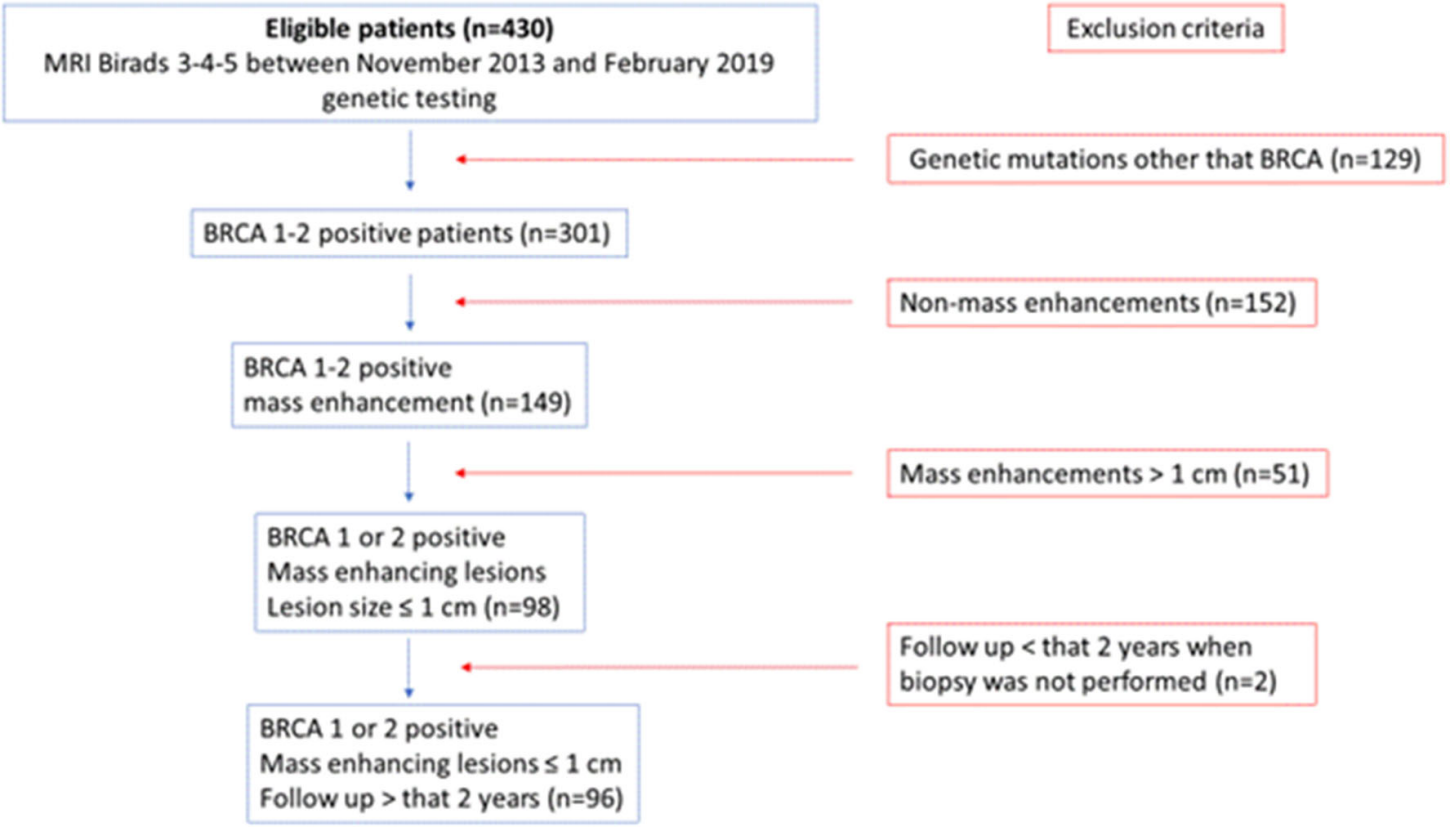
Flowchart of inclusion and exclusion criteria for the study

The study population included 96 patients (Fig. 1).

**Fig. 2:**
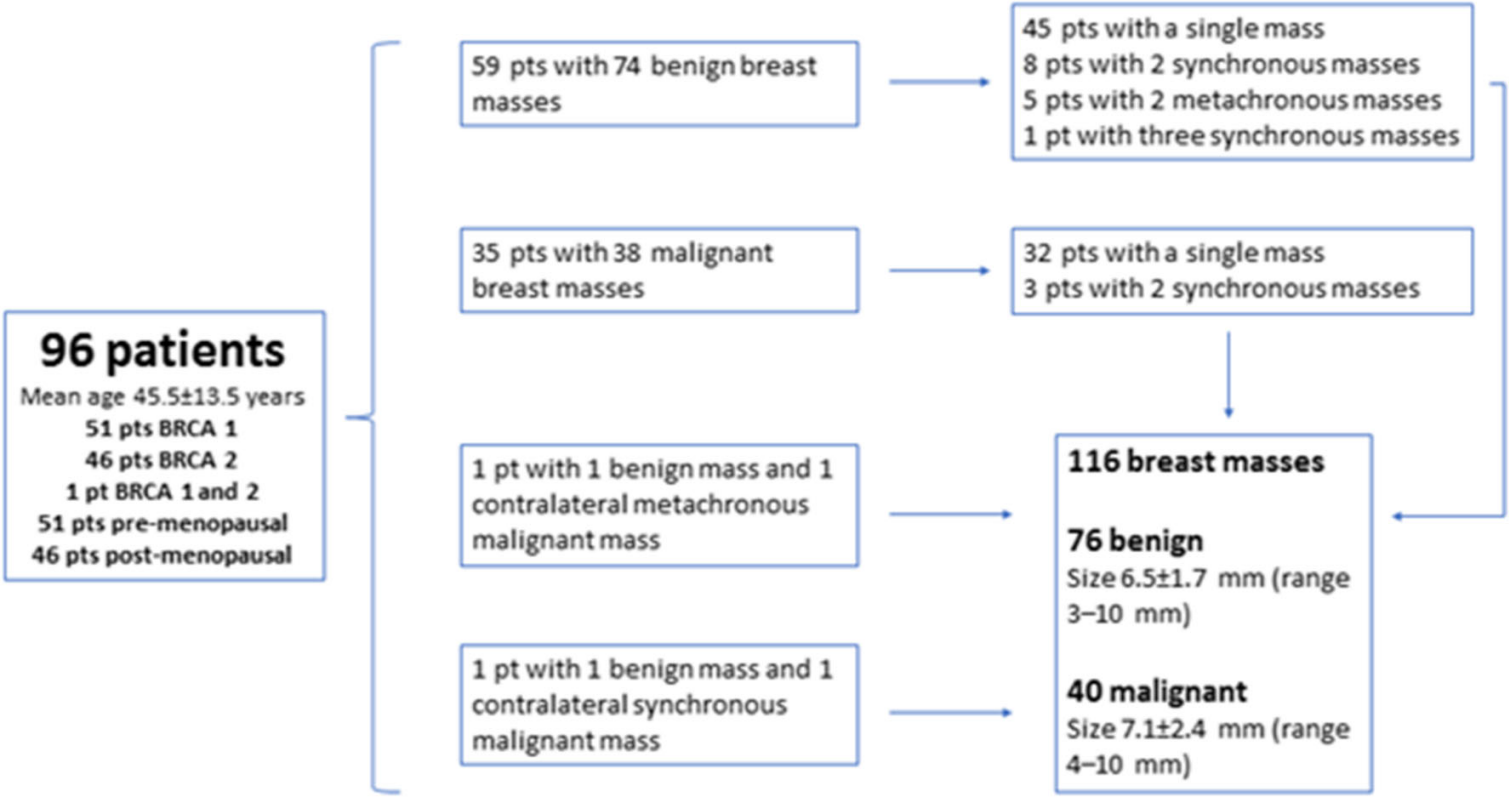
Patient and breast lesion characteristics

**Fig. 3:**
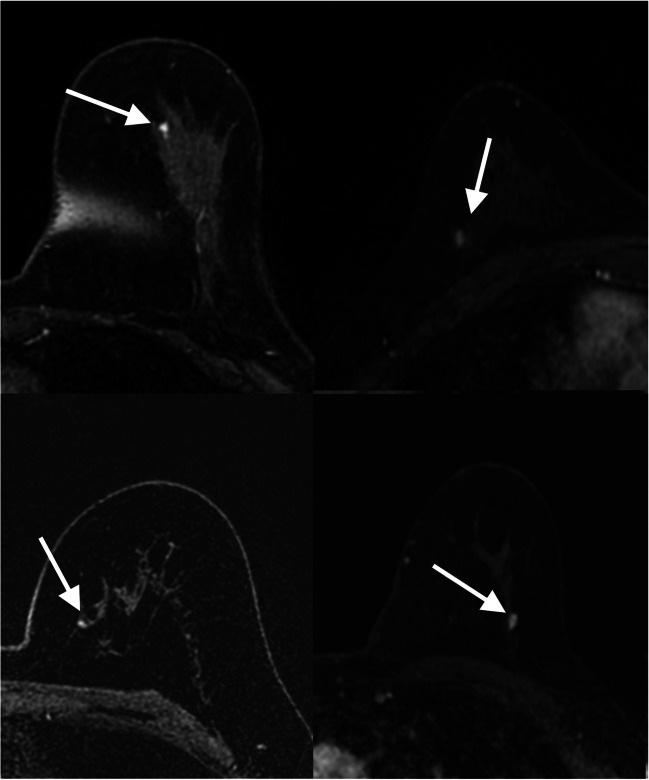
Transverse first post-contrast bilateral dynamic MR images (TR/TE, 4.5/2.1 ms; flip angle, 10°) of four patients with benign-appearing small breast masses (white arrows) in which biopsy yielded invasive ductal carcinoma

**Fig. 4:**
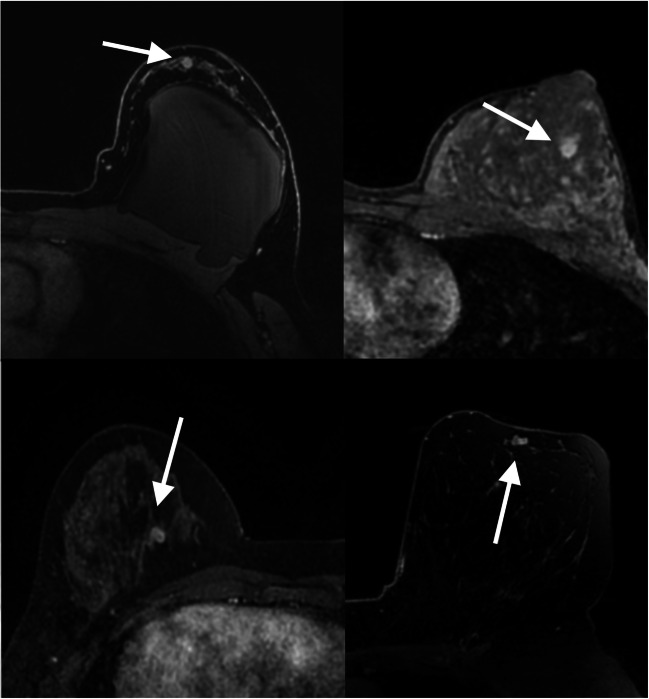
Transverse first post-contrast bilateral dynamic MR images (TR/TE, 4.5/2.1 ms; flip angle, 10°) of four patients with suspicious-appearing small breast masses categorized as BI-RADS 4 in which biopsy results yielded fibroadenoma (white arrows) and pseudoangiomatous stromal hyperplasia (white arrow)

Table 2 and Fig. 2 show the patient and breast lesion characteristics. Figures 3 and 4 are examples of benign and malignant breast masses included in this study. After segmentation, the median benign lesion size was 514.5 pixels (range 85–2425 pixels) and the median malignant lesion size was 816 pixels (range 66–2116 pixels).

**Table 2:** Histopathology of the 76 benign and 40 malignant masses

Benign (*n* = 76)	Malignant (*n* = 40)
FAD 21; complex FAD 1; FAD with atypia 1	IDC 29
Ruptured cyst, adenosis, stromal fibrosis and normal breast parenchyma 21	IDC + DCIS 4
PASH 12	ILC + DCIS 1
Papilloma 3	DCIS microinvasive 1
Usual ductal hyperplasia 2	DCIS 4
Fat necrosis 1	Metastatic intramammary lymph node 1
LCIS 1; ALH 1	
Columnar changes with atypia 1	
Benign follow-up (BI-RADS 3) 8	
Benign follow-up (BI-RADS 4), not visible at time of biopsy 3	

*FAD*, fibroadenoma; *PASH*, pseudoangiomatous stromal hyperplasia; *LCIS*, lobular carcinoma in situ; *ALH*, atypical lobular hyperplasia; *IDC*, invasive ductal carcinoma; *DCIS*, ductal carcinoma in situ

### Imaging assessment by radiologists

Consensus BI-RADS classification achieved a sensitivity of 75%, specificity of 42.1%, PPV of 40.5%, NPV of 76.2%, and accuracy of 53.4%. Time–intensity kinetic curve analysis was performed of 109/116 lesions; 7 lesions were not analyzed due to motion-related artifacts. Progressive contrast enhancement was present in 54.2% of patients with benign lesions (38/70) and in 23% of patients with malignant lesions (9/39); there was a statistically significant association with disease status based on kinetic analysis (*p* = 0.01).

Table 3 shows the results from univariate analysis according to independent assessments by the two radiologists.

**Table 3:** Univariate analysis according to independent radiologist assessment

	Reader 1			Reader 2		
Imaging feature	Malign.	Benign	*p* value	Malign.	Benign	*p* value
BI-RADS			0.003			0.002
2	1 (1)	1 (1)		3 (3)	12 (10)	
3	8 (7)	29 (25)		8 (7)	21(18)	
4	26 (22)	46 (40)		22 (19)	43 (37)	
5	5 (4)	0 (0)		7 (6)	0 (0)	
BPE			0.047			0.33
Minimal	20 (17)	20 (17)		23 (20)	35 (30)	
Mild	13(11)	33 (29)		10 (9)	25 (22)	
Moderate	5 (4)	14 (12)		5 (4)	8 (7)	
Marked	1 (1)	9 (8)		1 (1)	8 (7)	
Bilateral mastectomy	1			1		
Contrast enhancement			0.07			0.04
Homogeneous	11 (10)	25 (22)		10 (9)	17 (15)	
Heterogeneous	15(13)	14 (12)		17 (15)	16 (14)	
Rim enhancement	10 (9)	18 (16)		7 (6)	16 (14)	
Dark internal septations	4 (4)	19 (16)		6 (5)	27 (23)	
DWI signal			0.22			0.91
Homogeneous	12 (19)	19 (30)		9 (14)	16 (25)	
Heterogeneous	0 (0)	3 (5)		1 (2)	5 (8)	
Rim	2 (3)	1 (2)		1 (2)	3 (5)	
No correlation	7 (11)	21 (32)		10 (15)	20 (31)	
Margins			0.06			0.01
Circumscribed	22 (19)	54 (47)		18 (16)	48 (41)	
Irregular	16 (14)	22 (19)		18 (16)	28 (24)	
Spiculated	2 (2)	0 (0)		4 (3)	0 (0)	
Shape			0.19			0.03
Oval	11 (10)	34 (29)		13 (11)	36 (31)	
Round	15 (13)	21 (18)		11 (10)	27 (23)	
Irregular	14 (12)	21 (18)		16 (14)	13 (11)	
T2 signal intensity			0.02			0.17
Hypointense	6 (5)	4 (4)		3 (3)	v6 (5)	
Isointense	6 (5)	13 (11)		11 (10)	17 (15)	
Hyperintense	24 (21)	35 (30)		23 (20)	35 (30)	
No correlation	4 (4)	24 (21)		3 (3)	18 (16)	
FGT breast with mass			0.07			0.07
Almost entirely fat	5 (4)	2 (2)		5 (4)	9 (8)	
Scattered FGT	13 (11)	24 (21)		21 (18)	25 (22)	
Heterogeneous FGT	13 (11)	21 (18)		8 (7)	17 (15)	
Extreme FGT	8 (7)	29 (25)		5 (4)	25 (22)	
Mastectomy	1	0		1	0	
FGT contralateral breast			0.13			0.07
Almost entirely fat	3 (3)	1 (1)		3 (4)	8 (7)	
Scattered FGT	12 (11)	24 (22)		20 (18)	24 (22)	
Heterogeneous FGT	12 (11)	20 (18)		7 (6)	17 (16)	
Extreme FGT	8 (7)	29 (27)		5 (5)	25 (23)	
Mastectomy	5	2		5	2	

Values represent number of patients (percentages)

*BI-RADS*, Breast Imaging and Reporting and Data System; *BPE*, background parenchymal enhancement; *DWI*, diffusion-weighted imaging; *FGT*, fibroglandular tissue

Table 4 shows the results from univariate analysis according to overall consensus assessment as well as according to singular assessment performed for kinetics and lesion location, BRCA mutation status, and menopausal status. In consensus reading, there was no significant association with disease status based on margin (*p* = 0.11), shape (*p* = 0.97), enhancement pattern (*p* = 0.05), T2 signal intensity (*p* = 0.16), DWI (*p* = 0.54), BPE (*p* = 0.32), and BRCA mutation status (BRCA1 vs. BRCA2, *p* = 0.79). There was a statistically significant association with disease status based on lesion location within the breast (*p* = 0.03), menopausal status (*p* = 0.0001), and BI-RADS classification (*p* < 0.001).

**Table 4:** Consensus analysis according to independent radiologist assessment

	Disease status		
Imaging feature	Malignant	Benign	*p* value
BI-RADS			< 0.001
2	1 (1)	5 (4)	
3	9 (8)	27 (23)	
4	22 (19)	44 (38)	
5	8 (7)	0 (0)	
BPE			0.33
Minimal	23 (20)	35 (30)	
Mild	10 (9)	25 (22)	
Moderate	5 (4)	8 (7)	
Marked	1 (1)	8 (7)	
BRCA			0.80
1	20 (18)	38 (33)	
2	18 (16)	38 (33)	
Contrast enhancement			0.05
Homogeneous	9 (8)	18 (16)	
Heterogeneous	20 (17)	21(18)	
Rim enhancement	7 (6)	16 (14)	
Dark internal septation	4 (4)	21 (18)	
DCE (kinetics)*			0.01
Progressive	9 (8)	38 (35)	
Plateau	22 (20)	23 (21)	
Washout	8 (7)	9 (8)	
DWI signal			0.54
Homogeneous	10 (16)	19 (30)	
Heterogeneous	0 (0)	3 (5)	
Rim	2 (3)	2 (3)	
No correlation	8 (13)	19 (30)	
Location*			0.03
Anterior	5 (4)	20 (17)	
Middle	14 (12)	34 (29)	
Posterior	21 (18)	22 (19)	
Margins			0.11
Circumscribed	23 (20)	51 (44)	
Irregular	15 (13)	25 (22)	
Spiculated	2 (2)	0 (0.0)	
Menopausal status			< 0.001
Fertile	12 (10)	51 (44)	
Menopause	28 (24)	25 (22)	
Shape			0.97
Oval	14 (12)	28 (24)	
Round	14 (12)	25 (22)	
Irregular	12 (10)	23 (20)	
T2 Signal intensity			0.16
Hypointense	5 (4)	4 (4)	
Isointense	8 (7)	12 (10)	
Hyperintense	22 (19)	38 (33)	
No correlation	5 (4)	22 (19)	

Values represent number of patients (percentages)

*BI-RADS*, Breast Imaging and Reporting and Data System; *BPE*, background parenchymal enhancement; *DCE*, dynamic contrast-enhanced; *DWI*, diffusion-weighted imaging. *Evaluated by reader 1

### Radiomics analysis

#### ML Model using only the first post-contrast phase

At univariate analysis, 37/102 radiomics features were found to be significantly different between benign and malignant lesions (Supplemental Table S1). The AUC cutoff of ≥ 0.65 reduced the number of features of interest to 21/102. Correlation analysis resulted in 11 features (from 5 classes) for subsequent multivariate modeling (Supplemental Table S2). Using a fine Gaussian support vector machine with all 11 parameters, a perfect separation of benign and malignant cases was obtained, demonstrating 100% accuracy. However, this ML model undoubtedly overfitted the data (Supplemental Table S3).

After fivefold cross-validation, LASSO (least absolute shrinkage and selection operator) was used to further reduce the number of parameters. The final ML model utilized three parameters (GLCM-based correlation, SZM-based gray level non-uniformity normalized, and SZM-based zone emphasis). This ML model achieved a diagnostic accuracy of 75% but it can be regarded as a robust ML model which should produce similar results for new data (Supplemental Table S4). This ML model achieved a sensitivity of 55.0% (22/40), specificity of 85.5% (65/76), PPV of 66.7% (22/33), and NPV of 78.3% (65/83).

#### ML model combining radiomics features from the first post-contrast phase and clinical factors

We included clinical factors in multivariate modeling to further improve the model. Multivariate results showed that disease status was associated with menopausal status (*χ*^2^ = 11.86, *p* = 0.001), age (*p* < 0.0005), and lesion location (*χ*^2^ = 6.84, *p* = 0.03). There was no association with BRCA status (*χ*^2^ = 0.17, *p* = 0.68). A fivefold cross-validation was again employed to develop a robust ML model. The final ML model utilized six parameters (age, first-order coefficient of variation, GLCM-based joint entropy, GLCM-based correlation, GLCM-based cluster prominence, and RLM-based run emphasis). This robust ML model resulted in a diagnostic accuracy of 79.3% (Supplemental Table S5). This ML model achieved a sensitivity of 52.5% (21/40), specificity of 93.4% (71/76), PPV of 80.8% (21/26), and NPV of 78.9% (71/90).

#### ML model combining radiomics features from all dynamic phases and clinical factors

The results for the ML model using all dynamic phases and clinical factors are provided in the Supplemental Data (Supplemental Info A2, Table S6, Table S7, Table S8). This ML model resulted in a diagnostic accuracy of 81.5% and can be regarded as a robust model. The results from all radiomics models are illustrated in Table 5.

**Table 5:** Summary of radiomics features model results

	Accuracy	Sensitivity	Specificity	PPV	NPV
1st PC phase (no validation)	90.5% (83.7–95.2)	75.0% (58.8–87.3)	98.7% (92.9–100.0)	96.8% (80.9–99.5)	88.2% (81.4–92.8)
1st PC phase (fivefold validation)	75.0% (66.1–82.6)	55.0% (38.5–70.7)	85.5% (75.6–92.6)	66.7% (52.0–78.7)	78.3% (71.7–83.7)
1st PC phase and clinical factors (fivefold validation)	79.3% (70.8–86.3)	52.5% (36.1–68.5)	93.4% (85.3–97.8)	80.8% (63.1–91.2)	78.9 (72.9–83.9)
All phases and clinical factors (fivefold validation)	81.5% (72.9–88.3)	63.2% (46.0–78.2)	91.4% (82.3–96.8)	80.0% (64.2–89.9)	82.1% (75.0–87.5)

Confidence intervals are in parenthesis

*PC*, post-contrast; *PPV*, positive predictive value; *NPV*, negative predictive value

## Discussion

In this study, we investigated whether radiomics analysis and ML with MRI can accurately differentiate sub-centimeter benign from malignant lesions in BRCA mutation carriers using model-free parameter maps. We demonstrated that radiomics analysis coupled with ML aids in the differentiation of benign and malignant enhancing sub-centimeter masses in these patients. The T2-weighted signal intensity and DW imaging did not help to differentiate benign from malignant lesions. While larger cancers have been well-described and characterized on MRI, sub-centimeter lesions, particularly those less than 0.5 cm, have traditionally been regarded as being too small to characterize according to morphological descriptors, negatively impacting accuracy. With advancements in hardware and software, the spatial resolution of MRI has improved, allowing not only the detection but also the morphologic characterization of small enhancing lesions [19].

Meissnitzer et al [13] showed that sub-centimeter invasive breast cancers often present with benign morphologic features such as persistent enhancement (30%) and high T2 signal (17%). Raza et al [20] demonstrated that breast cancers smaller than 5 mm tend to present with circumscribed margins (71%), benign shape (67%), and benign kinetic characteristics (41%). The presence of a BRCA mutation is an additional confounding factor as breast cancers in this population often present with benign morphologic features (e.g., oval shape and well-defined margins) on MRI and can resemble a fibroadenoma or a cyst in 23–38% of cases [12, 20]. Yet, these cancers are more aggressive with fast growth rates and a short lead time [20].

Our results confirmed that for sub-centimeter masses in BRCA mutation carriers, morphologic BI-RADS descriptors are not particularly useful for breast cancer diagnosis; there was only moderate inter-rater agreement for morphology although there was at least substantial inter-rater agreement for the BI-RADS assessment categories. Compared with Ha et al [21] who concluded that any T2 hypointense enhancing focus representing an interval change should be biopsied rather than undergo short-term follow-up, we found no significant difference in T2 signal intensity between benign and malignant lesions. This is in agreement with Zhang et al who also showed that T2-weighted imaging does not significantly contribute to differentiating benign from malignant lesions [22]. In addition, we found that DWI signal analysis did not contribute to the accuracy of assessing these lesions, which can in part be explained by its limited spatial resolution which makes it challenging to accurately evaluate sub-centimeter masses.

Several studies have shown that radiomics and machine learning can be used as adjuvant tools to support radiologist image interpretation in differentiating benign from malignant lesions using mammography [23], digital breast tomosynthesis [24], and MRI [16, 25]. A study by Truhn et al [26] demonstrated that radiomics and CNN were superior compared with radiomics analysis in differentiating benign from malignant breast masses but both were inferior to the assessment performed by the radiologist. However, for this study, the authors included lesions with overall average diameter of 22.4 ± 20.3; thus, their results could be due to the fact that when lesions are larger in size, they are easier to be characterized as benign or malignant by just analyzing BI-RADS descriptors.

Our study shows a more accurate means of differentiating benign from malignant lesions in BRCA mutation carriers. Gibbs et al evaluated the utility of radiomics and ML from DCE-based parameter maps to diagnose small breast lesions in the general population [16]. The best AUC was 0.78 ± 0.12 and their results showed that radiomics can potentially improve the evaluation of small, benign-appearing breast masses, with increased PPV (fewer biopsies needed) and NPV (more cancers diagnosed) compared with the currently used BI-RADS classification alone. In our study population of BRCA mutation carriers, our data indicate that radiomics analysis and ML can in fact spare women from unnecessary biopsies for benign-appearing small breast nodules. Three radiomics features (coefficient of variation, cluster prominence, and Haralick correlation) were able to separate benign from malignant masses with a diagnostic accuracy of 79.3% when only the first post-contrast scan, combined with clinical data, was used in a ML model.

Another study by D’Amico et al [27] examined 12 malignant and 33 benign enhancing foci in 45 patients. From these foci, over 200 radiomics features were extracted and performances of selected features were evaluated by means of *k*-nearest neighbor (kNN). A fast and robust classification algorithm yielded a sensitivity of 27/27 (100%, 95% CI 87–100%), a specificity of 37/41 (90%, 95% CI 77–97%), and an accuracy of 64/68 (94%, 95% CI 86–98%). Compared with D’Amico et al, our study compared machine learning to radiologist’s image interpretation according to BI-RADS from 2 different readers, included a larger sample size of 116 lesions (vs. 45), and included a more homogeneous patient populations with BRCA mutations.

Recently, alternative abbreviated protocols have been proposed for screening women [19, 28] to reduce scan time by acquiring only one pre-contrast and one early post-contrast T1-weighted image set. In agreement with the results of Gibbs et al [16], our results showed that delayed post-contrast phases did not add any significant discriminative value to the analysis. This study therefore provides indirect evidence for the potential use of radiomics analysis in abbreviated protocols which have been recently proposed as an alternative for screening high-risk women with dense breast tissue [19] without concerns regarding a decrease in specificity related to the lack of information of enhancement kinetics in the delayed phases.

This study has limitations. By using only single-center data, it is difficult to predict how the developed models might perform with data acquired under different imaging protocols, especially in the case of poorer spatial resolution and slice thickness. We included only sub-centimeter breast masses which do not constitute many pixels in an image, leading to lower spatial resolution and fewer pixels in the final ROI and an increased proportion of pixels that can be regarded as potentially contaminated by partial volume effects. To ensure adequate counting statistics, we decreased the data to only 16 gray levels (vs. 32 or 64 gray levels that have previously been employed in breast MRI) [29]. Another limitation is the relatively small sample size of 116 breast masses due to our strict inclusion criteria. With only 40 cases in the malignant group, feature selection was performed prior to any cross-validation fold.

In conclusion, radiomics analysis coupled with machine learning improves the diagnostic accuracy in small breast masses in BRCA mutation carriers compared with the qualitative morphological assessment with BI-RADS classification alone. Further studies, preferentially multi-center studies in larger patient cohorts, are needed to confirm these promising results.

## Electronic supplementary material

ESM 1 (DOCX 38.7 kb)

## Abbreviations

BI-RADS: Breast Imaging Reporting and Data System
BPE: Background parenchymal enhancement
FGT: Fibroglandular tissue
GLCM: Gray level co-occurrence matrix
ML: Machine learning
NPV: Negative predictive value
PPV: Positive predictive value
RLM: Run length matrix
SZM: Size zone matrix
