# Supplementary material for: Improved characterization of sub-centimeter enhancing breast masses on MRI with radiomics and machine learning in BRCA mutation carriers
Source: Eur Radiol. 2020 Jun 27;30(12):6721–31. doi: 10.1007/s00330-020-06991-7 (PMC7599163; doi:10.1007/s00330-020-06991-7)
Supplement: Supplementary file 1 — (DOCX 38.7 kb) [file 330_2020_6991_MOESM1_ESM.docx]

**Supplemental Information**

**Supplemental Info A1: *Data Harmonization for Radiomics Data***

As patients were scanned at either 1.5T or 3T, ComBat [1] harmonization was employed prior to statistical analysis to remove field strength effects whilst retaining differences due to pathology. ComBat harmonization was initially described to remove batch effects in genomic data but has since successfully been applied to medical imaging data for the purposes of radiomics analysis [2]. Prior to data harmonization, 33/102 features exhibited significant differences due to field strength for either benign lesions, malignant lesions, or both. After data harmonization, only six features retained significant differences due to field strength.

1. Johnson WE, Li C, Rabinovic A (2007) Adjusting batch effects in microarray expression data using empirical Bayes methods. Biostatistics 8:118-127
2. Orlhac F, Boughdad S, Philippe C et al (2018) A Postreconstruction Harmonization Method for Multicenter Radiomic Studies in PET. J Nucl Med 59:1321-1328

**Table S1.** At univariate analysis, 37 features demonstrating significant differences between benign and malignant lesions

|  |  |  |  |  |
| --- | --- | --- | --- | --- |
| **Feature** | **Benign** | **Malignant** | ***p*-value** | **AUC (±SE)** |
|  | **(median and IQR)** | **(median and IQR)** |  |  |
| First Order Features | | | | |
| Coefficient of dispersion | 0.182 (0.135–0.214) | 0.195 (0.169−0.269) | 0.008 | 0.651 ± 0.052 |
| Coefficient of variation | 0.248 (0.193–0.290) | 0.274 (0.232−0.336) | 0.003 | 0.666 ± 0.051 |
|  |  |  |  |  |
| Gray Level Co-occurrence Matrix (GLCM)-Based Features | | | | |
| Energy (×10^-3^) | 5.5 (4.6–6.6) | 5.0 (3.9−6.1) | 0.044 | 0.614 ± 0.056 |
| Joint entropy | 7.92 (7.64–8.17) | 8.09 (7.95−8.30) | 0.002 | 0.677 ± 0.052 |
| Joint variance | 43.8 (38.7–49.8) | 46.4 (41.8−52.4) | 0.046 | 0.613 ± 0.054 |
| Sum variance | 157.7 (138.3–178.0) | 163.9 (153.6−187.2) | 0.01 | 0.646 ± 0.052 |
| Sum entropy | 5.46 (5.36–5.58) | 5.58 (5.47−5.64) | 0.002 | 0.677 ± 0.052 |
| Correlation | 0.760 (0.735–0.819) | 0.807 (0.775−0.831) | 0.001 | 0.688 ± 0.049 |
| Cluster tendency | 157.7 (138.3–178.0) | 163.9 (153.6−187.2) | 0.01 | 0.646 ± 0.052 |
| Cluster prominence (×10^3^) | 65.8 (53.7–76.6) | 72.7 (67.3−82.7) | 0.002 | 0.678 ± 0.050 |
| Haralick correlation (×10^4^) | 82.9 (64.0–110.6) | 109.3 (81.0−141.7) | 0.005 | 0.659 ± 0.052 |
| 1^st^ Correlation coefficient | −0.275 (−0.324 to −0.253) | −0.266 (−0.282 to −0.242) | 0.049 | 0.612 ± 0.054 |
| Run Length Matrix (RLM)-Based Features | | | | |
| Gray level non-uniformity | 17.8 (12.2–30.9) | 29.4 (18.9–46.8) | 0.004 | 0.663 ± 0.055 |
| Gray level non-uniformity normalized (×10^-2^) | 4.25 (4.04–4.70) | 4.05 (3.83–4.34) | 0.013 | 0.640 ± 0.054 |
|  |  |  |  |  |
| Gray level variance | 48.5 (43.8–53.9) | 52.4 (47.2–57.6) | 0.015 | 0.638 ± 0.053 |
| Long run high gray level emphasis | 554.4 ± 162.7 | 623.2 ± 179.2 | 0.039 | 0.608 ± 0.056 |
| Run emphasis | 5.085 (5.006–5.167) | 5.183 (5.118–5.281) | <0.0005 | 0.732 ± 0.049 |
| Run length non-uniformity | 367.1 (245.7–563.3) | 592.3 (384.5–900.1) | 0.001 | 0.683 ± 0.053 |
| Size Zone Matrix (SZM)-Based Features | | | | |
| Gray level non-uniformity | 12.7 (9.5–21.1) | 20.1 (13.8–31.3) | 0.003 | 0.669 ± 0.054 |
| Gray level non-uniformity normalized (×10^-2^) | 4.13 (3.94–4.44) | 3.92 (3.76–4.12) | 0.001 | 0.683 ± 0.052 |
| Size zone non-uniformity | 190.1 (130.2–286.7) | 285.3 (197.7–441.1) | 0.001 | 0.685 ± 0.052 |
| Large area low gray level emphasis (×10^-2^) | 3.78 (3.13–5.20) | 4.59 (3.52–6.28) | 0.033 | 0.621 ± 0.052 |
| Gray level variance | 48.0 (43.3–53.9) | 51.2 (47.2–55.6) | 0.01 | 0.647 ± 0.051 |
| Zone emphasis | 5.69 (5.50–5.84) | 5.86 (5.66–6.02) | 0.001 | 0.693 ± 0.053 |
| Neighborhood Gray Level Dependence Matrix (NGLDM)-Based Features | | | | |
| High dependence emphasis | 3.91 (3.17–4.74) | 4.36 (3.75–5.91) | 0.028 | 0.624 ± 0.054 |
| High dependence high gray level emphasis | 1830 (1293–2416) | 2289 (1544–3118) | 0.022 | 0.630 ± 0.055 |
| Gray level non-uniformity | 21.0 (13.6–34.3) | 34.8 (20.6–57.6) | 0.004 | 0.662 ± 0.055 |
| Gray level non-uniformity normalized (×10^-2^) | 4.30 (4.06–4.78) | 4.10 (3.81–4.44) | 0.038 | 0.617 ± 0.055 |
| Dependence count non-uniformity | 183.4 (131.0–281.5) | 267.9 (191.4–412.9) | 0.002 | 0.679 ± 0.052 |
| Gray level variance | 48.8 (42.8–53.6) | 52.6 (46.5–58.2) | 0.019 | 0.633 ± 0.053 |
| Dependence count variance | 0.898 (0.602–1.176) | 1.068 (0.868–1.556) | 0.016 | 0.637 ± 0.053 |
| Entropy | 5.99 (5.78–6.22) | 6.22 (6.05–6.38) | < 0.0005 | 0.710 ± 0.051 |
| Energy (×10^-2^) | 1.83 (1.61–2.12) | 1.60 (1.44–1.78) | < 0.0005 | 0.717 ± 0.051 |
| Neighborhood Grey Tone Difference Matrix (NGTDM)-Based Features | | | | |
| Coarseness (×10^-2^) | 3.14 (2.13–4.25) | 2.13 (1.53–3.37) | 0.006 | 0.655 ± 0.053 |
| Busyness (×10^-2^) | 6.66 (4.90–9.90) | 9.28 (6.38–13.94) | 0.005 | 0.658 ± 0.053 |
| Complexity | 630.2 (541.4–758.3) | 577.0 (503.2–630.4) | 0.012 | 0.642 ± 0.052 |
| Strength | 10.22 (6.92–15.19) | 6.65 (4.97–11.22) | 0.006 | 0.655 ± 0.053 |
|  |  |  |  |  |
|  |  |  |  |  |

**Table S2.** From the 37 significant features in Table S1, applying an AUC cut-off ≥ 0.65 and performing correlation analysis to remove redundant features resulted in 11 features demonstrating significant differences between benign and malignant lesions

| **Texture Class** | **Feature** | ***p*-value** | **AUC (±SE)** |
| --- | --- | --- | --- |
| First Order | Coefficient of variation | 0.003 | 0.666±0.051 |
| GLCM | Joint entropy | 0.002 | 0.677±0.052 |
| GLCM | Sum entropy | 0.002 | 0.677±0.052 |
| GLCM | Correlation | 0.001 | 0.688±0.049 |
| GLCM | Cluster prominence (×10^3^) | 0.002 | 0.678±0.050 |
| GLCM | Haralick correlation (×10^4^) | 0.005 | 0.659±0.052 |
| RLM | Run emphasis | <0.0005 | 0.732±0.049 |
| SZM | Gray level non-uniformity normalized (×10^-2^) | 0.001 | 0.683±0.052 |
| SZM | Size zone non-uniformity | 0.001 | 0.685±0.052 |
| SZM | Zone emphasis | 0.001 | 0.693±0.053 |
| NGTDM | Busyness (×10^-2^) | 0.005 | 0.658±0.053 |


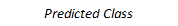
**Table S3.** Confusion matrix for the radiomics model with three features (coefficient of variation, cluster prominence, and Haralick correlation)

|  | Benign | Malignant |
| --- | --- | --- |
| Benign | 75 | 1 |
| Malignant | 10 | 30 |


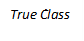


**Table S4.** Confusion matrix for the radiomics model following five-fold cross validation with three features (GLCM-based correlation, SZM-based gray level non-uniformity normalized, and SZM-based zone emphasis)


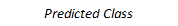


|  | Benign | Malignant |
| --- | --- | --- |
| Benign | 65 | 11 |
| Malignant | 18 | 22 |


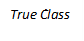


**Table S5.** Confusion matrix for the model combining radiomics features from the first post-contrast phase and clinical factors following five-fold cross validation with six parameters (age, first order coefficient of variation, GLCM-based joint entropy, GLCM-based correlation, GLCM-based cluster prominence, and RLM-based run emphasis)


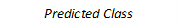


|  | Benign | Malignant |
| --- | --- | --- |
| Benign | 71 | 5 |
| Malignant | 19 | 21 |


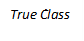


**Supplemental Info A2: *ML Model Combining Radiomics Features from All Dynamic Phases and Clinical Factors***

We also analyzed radiomic features from all four dynamic phases (pre-contrast, first post-contrast, second post-contrast, and third post-contrast). After careful visual inspection of the ROIs across all contrast enhancement phases, cases with gross motion artifacts were eliminated. There were 70 benign and 38 malignant lesions available for analysis. (See Supplemental Table S6 for the significant radiomic features for each phase).

Correlation analysis across all four phases reduced the number of radiomic features from 18 (3+12+1+2) to 12 radiomic features (See Supplemental Table S7 for the list of significant radiomic features). Five-fold cross validation was employed to develop a robust ML model incorporating radiomic features as well as clinical factors. The final ML model used only five parameters (age, lesion location, GLCM-based correlation from the pre-contrast phase, first-order coefficient of variation from the 1st post-contrast phase, and SZM-based gray level variance from the 1st post-contrast phase). This ML model resulted in a diagnostic accuracy of 81.5% and can be regarded as a robust model. (See Supplemental Table S8 for the confusion matrix.) This ML model achieved a sensitivity of 63.2% (24/38), a specificity of 91.4% (64/70), a PPV of 80.0% (24/30) and an NPV of 82.1% (64/78).

**Table S6.** Significant radiomic features for each dynamic phase

| **Parameter** | **Benign**  **(mean±SD or**  **median and IQR)** | **Malignant**  **(mean±SD or**  **median and IQR)** | ***p*-value** | **AUC (±SE)** |
| --- | --- | --- | --- | --- |
| Pre-contrast phase | | | | |
| Correlation (GLCM) | 0.806±0.063 | 0.847±0.044 | <0.0005 | 0.688±0.051 |
| Run length non-uniformity (RLM) | 358 (235-547) | 534 (329-905) | 0.007 | 0.666±0.056 |
| Entropy (NGLDM) | 6.048 (5.855-6.215) | 6.270 (6.027-6.356) | 0.002 | 0.682±0.055 |

| First post-contrast phase | | | | |
| --- | --- | --- | --- | --- |
| Coefficient of variation (FO) | 0.241 (0.191–0.277) | 0.264 (0.229–0.339) | 0.004 | 0.667±0.052 |
| Joint entropy (GLCM) | 7.92 (7.67–8.15) | 8.07 (7.93–8.25) | 0.004 | 0.667±0.054 |
| Correlation (GLCM) | 0.774 (0.753–0.823) | 0.811 (0.711–0.835) | 0.004 | 0.666±0.053 |
| Cluster tendency (GLCM) | 155.1 (137.8–174.9) | 170.9 (152.8–191.1) | 0.004 | 0.668±0.053 |
| Cluster prominence (×10^3^) (GLCM) | 65.7 (52.6–75.4) | 74.4 (67.8–86.9) | 0.002 | 0.707±0.050 |
| Haralick correlation (×10^4^) (GLCM) | 85.7 (66.2–109.1) | 104.9 (78.8–141.2) | <0.0005 | 0.656±0.054 |
| Run emphasis (RLM) | 5.115 (5.029–5.177) | 5.175 (5.099–5.291) | 0.001 | 0.692±0.054 |
| Gray level non-uniformity normalized (×10^-2^) (SZM) | 4.13 (3.94–4.48) | 3.96 (3.73–4.15) | 0.003 | 0.676±0.054 |
| Size zone non-uniformity (SZM) | 202.2 (126.6–289.9) | 287.6 (180.0–439.8) | 0.004 | 0.670±0.055 |
| Gray level variance (SZM) | 47.1 (42.6–52.9) | 52.6 (46.7–55.8) | 0.005 | 0.664±0.053 |
| Zone emphasis (SZM) | 5.71 (5.56–5.85) | 5.83 (5.65–5.99) | 0.007 | 0.658±0.057 |
| Busyness (×10^-2^) (NGTDM) | 6.68 (4.92–9.16) | 8.82 (6.58–14.61) | 0.007 | 0.658±0.055 |

| Second post-contrast phase | | | | |
| --- | --- | --- | --- | --- |
| Gray level non-uniformity (NGLDM) | 23.8 (14.4–36.8) | 37.8 (19.8–66.0) | 0.004 | 0.67±0.056 |

| Third post-contrast phase | | | | |
| --- | --- | --- | --- | --- |
| Gray level non-uniformity (RLM) | 20.41 (12.76-32.40) | 33.71 (17.90-53.81) | 0.004 | 0.67±0.056 |
| Zone emphasis (SZM) | 5.746 (5.549-5.929) | 5.915 (5.731-6.013) | 0.01 | 0.65±0.054 |

**Table S7.** Significant radiomic features across all dynamic phases after correlation analysis to reduce redundant features

| **Dynamic Phase** | **Parameter** | ***p*-value** | **AUC ( ± SE)** |
| --- | --- | --- | --- |
| Pre | Correlation (GLCM) | < 0.001 | 0.688 ± 0.051 |
| Pre | Entropy (NGLDM) | 0.002 | 0.682 ± 0.055 |
| 1^st^ Post | Coefficient of variation (First Order) | 0.004 | 0.667 ± 0.052 |
| 1^st^ Post | Joint entropy (GLCM) | 0.004 | 0.667 ± 0.054 |
| 1^st^ Post | Correlation (GLCM) | 0.004 | 0.666 ± 0.053 |
| 1^st^ Post | Cluster tendency (GLCM) | 0.004 | 0.668 ± 0.053 |
| 1^st^ Post | Cluster prominence (GLCM) | 0.002 | 0.707 ± 0.050 |
| 1^st^ Post | Haralick correlation (GLCM) | < 0.001 | 0.656 ± 0.054 |
| 1^st^ Post | Run emphasis (RLM) | 0.001 | 0.692 ± 0.054 |
| 1^st^ Post | Gray level non-uniformity normalized (SZM) | 0.003 | 0.676 ± 0.054 |
| 1^st^ Post | Size zone non-uniformity (SZM) | 0.004 | 0.670 ± 0.055 |
| 1^st^ Post | Gray level variance (SZM) | 0.01 | 0.664 ± 0.053 |
| 1^st^ Post | Zone emphasis (SZM) | 0.01 | 0.658 ± 0.057 |
| 1^st^ Post | Busyness (NGTDM) | 0.01 | 0.658 ± 0.055 |
| 3^rd^ Post | Zone emphasis (SZM) | 0.01 | 0.651 ± 0.054 |

**Table S8.** Confusion matrix for the model combining radiomics features from all dynamic phases and clinical factors


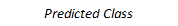


|  | Benign | Malignant |
| --- | --- | --- |
| Benign | 64 | 14 |
| Malignant | 6 | 24 |


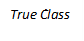


1
